# Laminin-511 Activates the Human Induced Pluripotent Stem Cell Survival via α6β1 Integrin-Fyn-RhoA-ROCK Signaling

**DOI:** 10.1089/scd.2022.0010

**Published:** 2022-11-08

**Authors:** Yoshiki Nakashima, Masayoshi Tsukahara

**Affiliations:** Kyoto University Center for iPS Cell Research and Application Foundation (CiRA Foundation), Facility for iPS Cell Therapy (FiT), Kyoto, Japan.

**Keywords:** Laminin-511, human induced pluripotent stem cells, signaling pathway, ROCK inhibitor (Y-27632)

## Abstract

In human induced pluripotent stem cells (hiPSCs), laminin-511/α6β1 integrin interacts with E-cadherin, an intercellular adhesion molecule, to induce the activation of the phosphatidylinositol 3-kinase (PI3K)-dependent signaling pathway. The interaction between laminin-511/α6β1 integrin and E-cadherin, an intercellular adhesion molecule, results in protection against apoptosis through the proto-oncogene tyrosine-protein kinase Fyn(Fyn)-RhoA-ROCK signaling pathway and the Ras homolog gene family member A (RhoA)/Rho kinase (ROCK) signaling pathway (the major pathway for cell death). In this article, the impact of laminin-511 on hiPSC on α6β1 integrin-Fyn-RhoA-ROCK signaling is discussed and explored along with validation experiments. *PIK3CA* mRNA (mean [standard deviation {SD}]: iMatrix-511, 1.00 [0.61]; collagen+MFGE8, 0.023 [0.02]; **P < 0.01; *n* = 6) and *PIK3R1* mRNA (mean [SD]: iMatrix-511, 1.00 [0.79]; collagen+MFGE8, 0.040 [0.06]; *P < 0.05; *n* = 6) were upregulated by iMatrix-511 resulting from an increased expression of *Integrin α6* mRNA (mean [SD]: iMatrix-511, 1.00 [0.42]; collagen+MFGE8, 0.23 [0.05]; **P < 0.01; *n* = 6). The iMatrix-511 increased the expression of p120-*Catenin* mRNA (mean [SD]: iMatrix-511, 1.00 [0.71]; collagen+MFGE8, 0.025 [0.03]; **P < 0.01; *n* = 6) and *RAC1* mRNA (mean [SD]: iMatrix-511, 1.00 [0.28]; collagen+MFGE8, 0.39 [0.15]; **P < 0.01; *n* = 6) by increasing the expression of *E-cadherin* mRNA (mean [SD]: iMatrix-511, 1.00 [0.38]; collagen+MFGE8, 0.16 [0.11]; **P < 0.01; *n* = 6). As a result, iMatrix-511 increased the expression of P190 *RhoGAP* (*GTPase-activating proteins*) mRNA, such as *ARHGAP1* mRNA (mean [SD]: iMatrix-511, 1.00 [0.57]; collagen+MFGE8, 0.032 [0.03]; **P < 0.01; *n* = 6), ARHGAP4 mRNA (mean [SD]: iMatrix-511, 1.00 [0.56]; collagen+MFGE8, 0.039 [0.049]; **P < 0.01; *n* = 6), and *ARHGAP5* mRNA (mean [SD]: iMatrix-511, 1.00 [0.39]; collagen+MFGE8, 0.063 [0.043]; **P < 0.01; *n* = 6). Western blotting showed that phospho-Rac1 remained in the cytoplasm and phospho-Fyn showed nuclear transition in iPSCs cultured on iMatrix-511. Proteome analysis showed that PI3K signaling was enhanced and cytoskeletal actin was activated in iPSCs cultured on iMatrix-511. In conclusion, laminin-511 strongly activated the cell survival by promoting α6β1 integrin-Fyn-RhoA-ROCK signaling in hiPSCs.

## Introduction

In 2016, a new type of induced pluripotent stem cell (iPSC) culture medium (named StemFit) [1] was developed in a joint venture between Professor Yamanaka of Kyoto University (the creator of iPSCs) and Ajinomoto Co., Ltd. The laminin-511 scaffold is capable of binding the α3β1, α6β1, and α6β4 integrins and maintaining hPSCs in an undifferentiated state in serum-free and xeno-free medium under feeder cell-free culture conditions [2–6]. StemFit medium uses laminin-511 as a scaffold material for cells and is currently the standard medium for culturing human iPSCs (hiPSCs) for medical use in Japan. At the time, we described in detail the intracellular signaling pathways at work in the culture conditions of these revolutionary clinical iPSCs [7]. At present, >6 years after its development, no clinical culture material has been developed that surpasses the combination of StemFit and laminin-511 as a scaffold material. In this study, we attempted to identify the effects of laminin-511 scaffolds by examining their effects on signaling pathways.

Laminin-511-based culture systems were found to inhibit cell death through α6β1 integrin-Fyn-RhoA-ROCK signaling. However, because trypsin (used during cell passaging) transiently cleaves the binding of the laminin-511 scaffold to α6β1 integrin in the StemFit–laminin-511 culture system and blocks the cell signaling pathway, iPSCs require a localized ROCK inhibitor (Y-27632) during the passaging process. Furthermore, the repair of trypsin-cleaved laminin-511 scaffold binding to α6β1 integrin requires 24 h after passaging [8]. In this study, we meticulously detail the effects of the laminin-511 scaffold and the α6β1 integrin-Fyn-RhoA-ROCK signaling pathway on hiPSC culture operations.

## Materials and Methods

### Reagents

StemFit AK03N was obtained from AJINOMOTO HEALTHY SUPPLY CO., INC. (Tokyo, Japan). The iMatrix-511 was obtained from Matrixome, Inc. (Osaka, Japan). A 10-mM Y-27632 Solution, D-PBS (−), and 0.5 M-EDTA Solution (pH 8.0) were obtained from Nacalai Tesque (Kyoto, Japan). TrypL™ Select Enzyme (1 × ) was obtained from Thermo Fisher Scientific K.K. (Kanagawa, Japan). Recombinant human MFGE8, Anti-Human E-Cadherin Affinity Purified Polyclonal Ab, and Goat IgG HRP-conjugated Antibody were purchased from R&D Systems (Minneapolis, MN). Anti-beta-actin (C4), Anti-Fyn (D-1), phospho Thr12, Anti-Rock-1 (B-1), and Anti-Rock-2 (D-11) were purchased from Santa Cruz Biotechnology, Inc., (Dallas, TX). GLS250 Gelatin Solution (1.0 mg/g) was purchased from Nitta Gelatin, Inc. (Osaka, Japan). Anti-Integrin α6-CD49f Antibody clone 5I3 ZooMAb(R), Anti-Integrin β1 Antibody clone 1A27 ZooMAb(R), and Anti-phospho-RAC1 (pSer71) antibody were purchased from Sigma-Aldrich Co. LLC (St. Louis, MO).

Fyn Antibody, p190-B RhoGAP Antibody and p190-A RhoGAP Antibody, Anti-rabbit IgG, HRP-linked Antibody, Anti-mouse IgG, and HRP-linked Antibody were purchased from Cell Signaling Technology, Inc., (Danvers, MA). Anti Rac1, Anti p120 Catenin, and Anti RHOA were purchased from Proteintech Group, Inc., (Rosemont, IL). Anti-PI3-kinase (p85α) mAb was purchased from Medical & Biological Laboratories Co., Ltd. (Tokyo, Japan). Anti-PI3 Kinase Antibody, p110α was purchased from Merck & Co., Inc. (Land Hessen, Germany). Goat Anti-Mouse IgM HRP Conjugate was purchased from Tokyo Chemical Industry Co., Ltd. (Tokyo, Japan).

### Maintenance culture of human induced pluripotent stem cells

The hiPSC line 201B7 was established by Shinya Yamanaka (CiRA Foundation) and obtained from CiRA Foundation (Kyoto, Japan). To culture iPSCs, a publicly available method (CiRA_Ff-iPSC_protocol_Eng_v140310) was used (https://www.cira.kyoto-u.ac.jp/j/research/img/protocol/Ff-iPSC-culture_protocol_E_v140311.pdf). Collagen coating was performed by adding 200 μL of GLS250 Gelatin Solution (1.0 mg/g) and 100 μL (5 μg) of recombinant human MFGE8 (50 μg/mL) to one well of a six-well plate.

### Real-time PCR

RNA was prepared using a SuperPREP II Cell Lysis & RT Kit for quantitative PCR (TOYOBO CO., LTD., Osaka, Japan) according to the manufacturer's instructions. Real-time PCR was performed using a StepOnePlus system (Life Technologies, Carlsbad, CA). Luna Universal qPCR Master Mix (New England Biolabs, Inc.) was used according to the manufacturer's instructions. For the design of human β-actin, human integrin α6, human integrin β1, and human cadherin 1 (CDH1) primers, the gene names were retrieved from the U.S. National Library of Medicine NIH website. The human β-actin, human integrin α6, human integrin β1, and human cadherin 1 (CDH1) primers were designed using the Primer 3 Plus application. Other primers were purchased from TaKaRa Bio, Inc. (Shiga, Japan). The primers used for PCR were as follows:

human integrin α6 (NM_000210.3) 195 bp
(forward) GTTTTGTTTCCTCCCCTATCTGTAT
(reverse) GCTCCCCATATAACTTAACATTGTG
human integrin β1 (NM_002211.3) 179 bp
(forward) CTGAAGACTATCCCATTGACCTCTA
(reverse) GCTAATGTAAGGCATCACAGTCTTT
human cadherin 1 (CDH1) (NM_004360.4) 191 bp
(forward) GCCACATCTTGACTAGGTATTGTCT
(reverse) GCAGCACTTTAGGCACTATTCTAAG
human β-actin (NM_001101.3) 224 bp
(forward) GTGACATTAAGGAGAAGCTGTGCTA
(reverse) CTTCATGATGGAGTTGAAGGTAGTT
HA348692 (FYN)
(forward) CAATTCCGTAGCCAGCTGCTC
(reverse) CCATGGAAGTTCGTCAGCTTCA
HA370128 (PIK3CA)
(forward) TCAGTCCTCAGACCTAATTGGGTTG
(reverse) TCTTGATAMGCTTCCAGGTGAACA
HA377172 (PIK3R1)
(forward) AGCATTGGGACCTCACATTACACA
(reverse) TACTGGAAACACAGTCCATGCACA
HA320879 (CTNND1)
(forward) TGCTCCTGATGATGGTCCTGTC
(reverse) CCTGGTCTCACTAGCCCATGAA
HA356749 (RAC1)
(forward) CCTGTAGTCGCTTTGCCTATTGA
(reverse) AGGGTCCCACGCTGTATTCTC
HA356425 (ARHGAP1)
(forward) CCGAGAGCTACAGCGTGTGA
(reverse) GGGCCAGTGTGACTCCGTAA
HA144198 (ARHGAP4)
(forward) GCAACACGTGGAGGTGGATAA
(reverse) GTGGTAGATGCTGGCCCAAG
HA134083 (ARHGAP5)
(forward) TTTGAGCTGTGGCTAGACATTCTT
(reverse) GACCTCACCAGATCCAGACTGAC
HA390884 (RHOA)
(forward) CAGCTGCMGGTACTCTGGTGA
(reverse) CTCTGCCACAGCTGCATGAA
HA271276 (ROCK1P1)
(forward) ACAAATATCACAGGCTTCAGGGTTA
(reverse) TGTAGGCAAACCCGCGATA
HA355736 (ROCK1)
(forward) TGCAACTGGAACTCAACCAAGAA
(reverse) GCTGGCCAACTGCATCTGAA
HA359025 (ROCK2)
(forward) GCAAGTCACTGCCGAGCTTC
(reverse) GCTGTCACACAGTGCTTATGTTCA

### Western blotting

Western blotting analyses using ATTO Products, EzRIPA Lysis kit, EzSubcell Extract, cPAGE Twin, myPower II 300, HorizeBLOT 2M-R, EzApply, EzStandard PrestainBlue, c-PAGEL 10%, EzRun, P plus membranes, Filter paper, EzFastBlot, EzBlock Chemi, EzTBS, and EzWestBlue, were performed according to the manufacturer's instructions (ATTO, Tokyo, Japan). Blots were probed using specific antibodies for E-Cadherin, integrin α6, integrin β1, Fyn, phospho-Fyn, Rac1, phospho-Rac1, p120 catenin, PI3-kinase (p85α), PI3-kinase (p110α), p190-A RhoGAP, p190-B RhoGAP, RhoA, ROCK-1, ROCK-2, and β-actin.

### Standard data-independent acquisition proteome analyses

The following sample preparation procedures were performed as pretreatment for the proteome analysis: (1) Add chloroform to the sample containing TRIzol, mix, and centrifuge (15,000*g*, 4°C, 15 min). (2) Remove the aqueous layer, add acetonitrile to the remaining TRIzol solution, and precipitate the protein. (3) Add 100 mM Tris-HCL pH 8.5, 2% SDS to the precipitate, and dissolve the protein using a sealed ultrasonic disruption machine. (4) Measure the protein concentration by a bicinchoninic acid (BCA) assay, adjusted with 100 mM Tris-HCL pH8.5, 2% SDS to make protein concentration 0.5 μg/μL. (5) Cleave the S-S bond of the protein by adding Tris(2-carboxyethyl)phosphine to the protein lysate (20 μg protein) to a final concentration of 20 mM, and then incubate at 80°C for 10 min. (6) Alkylate cysteine residues by adding iodoacetamide to a final concentration of 30 mM, and then incubate at room temperature (light shielded) for 30 min. (7) Mix Sera-Mag SpeedBead Carboxylate-Modified Magnetic Particles (Hydrophylic) from Cytiva and Sera-Mag Carboxylate-Modified Magnetic Particles (Hydrophobic) at a 1:1 (v/v) ratio and wash three times with distilled water to 15 μg solids/μL in distilled water (SP3 beads). (8) Put 20 μL of SP3 beads into the alkylated sample, add another 2.5 times the sample liquid volume of ethanol, and mix at room temperature for 20 min. (9) After washing the beads twice with 80% ethanol, add 100 μL of 50 mM Tris-HCL pH 8.0 and mix. (10) Add 500 ng of Trypsin/Lys-C Mix (Promega) for peptide fragmentation and incubate at 37°C overnight. (11) Add 20 μL of 5% trifluoroacetic acid (TFA) and process in a sample-sealing ultrasonic disruption machine. (12) Desalt using a reversed-phase spin column (GL-Tip SDD; GL Sciences) and dry using a centrifugal evaporator. (13) Add 2% acetonitrile (ACN)-0.1% TFA and dissolve the peptides in a sample sealed sonication system. (14) Measure the peptide concentration by a BCA assay and adjust with 2% ACN-0.1% TFA to reach 200 ng/μL peptide concentration. (15) Conduct an LC-MS analysis.

The prepared samples were subjected to nanoLC-MS/MS under appropriate analytical conditions, as follows: injected peptide volume, 300 ng; nanoLC used, UltiMate 3000 RSLCnano LC System (Thermo Fisher Scientific); Column size, 75 μm i.d. × 120 mm length (Nikkyo Technos); column temperature, 40°C; solvents, Solvent A, distilled water with 0.1% formic acid; Solvent B, 80% ACN with 0.1% formic acid.

The MS data obtained were analyzed using Scaffold data-independent acquisition (DIA) under the following conditions: software program used, Scaffold DIA (Proteome Software); Protein Sequence Database, Human UniProtKB/Swiss-Prot database (Proteome ID UP000005640, 20588 entries, downloaded on November 26, 2021); Spectral Library, created by Prosit (https://www.proteomicsdb.org/prosit/) from the above sequence database.

Data analyses were performed in Perseus (https://maxquant.net/perseus/). The quantitative values of the proteome analysis results were Log2 transformed and missing values (quantitative values of 0) were randomly assigned at low values that were below the detection limit.

### Statistical analyses

Statistical analyses were performed using Student's *t*-test to compare two samples. *P* values of <0.05 were considered statistically significant for all tests.

### Institutional review board

This research does not involve human subjects, and does not need to obtain Institutional Review Board (IRB).

## Results

### Elucidation of the signaling pathways involved in the process of promoting iPSC viability in laminin-511 culture

**FIG. 1.**
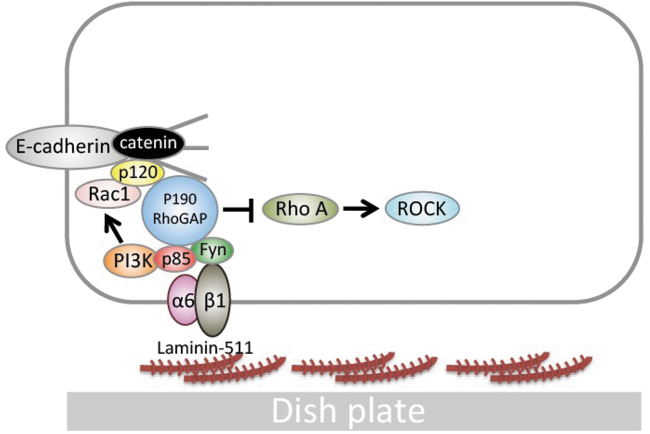
Fyn-RhoA-ROCK signaling (on laminin-511). In cultures using laminin-511 scaffolds, the E-cadherin-α6β1 integrin signaling pathway regulates hiPSC cell death. PI3K signaling promotes the expression of Rac1, which binds to cadherin, inhibiting cadherin endocytosis and increasing the expression of cadherin on the plasma membrane. When α6β1 integrin binds to laminin-511, Fyn-RhoA-ROCK signaling is induced. As a result, ROCK-induced hiPSC cell death is inhibited. This figure is a modified version of an illustration in our previous article [7]. hiPSC, human induced pluripotent stem cells; PI3K, phosphatidylinositol 3-kinase.

Laminin-511, a scaffolding component composed of α5, β1, and γ1 chains [9], binds to integrin**s** on the cell membrane (α3β1 [10], α6β1 [11], and α6β4 [12]), which induces the activation of the phosphatidylinositol 3-kinase (PI3K)/AKT-dependent [13] and Ras/MAPK-dependent signaling pathways [7]. In hiPSCs, the interaction between laminin-511/α6β1 integrin and E-cadherin (a cell–cell adhesion molecule) provides protection against apoptosis through the proto-oncogene tyrosine-protein kinase Fyn (Fyn)-RhoA-ROCK signaling pathway [7] and the Ras homolog gene family member A (RhoA)/Rho kinase (ROCK) signaling pathway, which is the major pathway for cell death [14] (Fig. 1). An older scaffold material that does not use iMatrix-511 is gelatin. Gelatin was used in the feeder culture method with mouse embryonic fibroblasts (MEFs) [14]. In addition, we have previously reported that MFGE8 (Milk Fat Globule EGF and Factor V/VIII Domain Containing), secreted by MEFs themselves, promotes the cellular adhesion of hiPSCs [15].

**FIG. 2.**
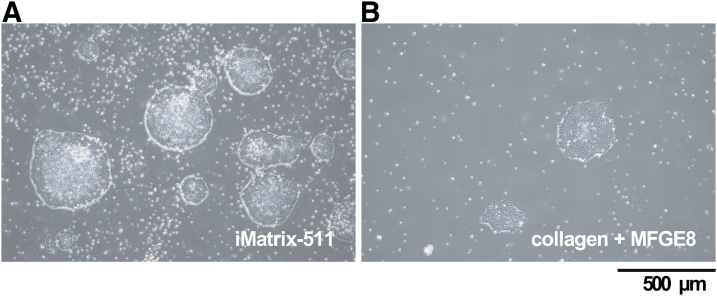
Cell observation. Photographs of hiPSCs taken by optical microscope at 6 days after seeding on each well coated with iMatrix511 **(A)** or collagen+MFGE8 **(B)**. Scale bar = 500 μm.

Therefore, we chose to use gelatin+MFGE8 as a scaffold material, which is the least likely to affect the intracellular signaling pathways, as a control for iMatrix-511 in this study. As a result, in comparison to iMatrix-511, colonies were observed in gelatin+MFGE8, similarly to the ES cells of iPSCs, but there were fewer colonies. This result indicates that iMatrix-511 has the effect of promoting cell survival activity (Fig. 2).

### Laminin receptors (integrin α6β1 and α6β4)

PSCs interact with laminin-511 through β1-integrins (predominantly α6β1). α6β1 has broad specificity and is capable of binding to a number of laminin isoforms. However, laminin-511 and laminin-521 are the only isoforms that maintain the pluripotency of hPSCs after α6β1 integrin signaling and thereby induce the PI3K/AKT signaling pathway [16,17]. In hPSCs, laminin-511 prevents the induction of apoptosis by ROCK in vitro. In cultures that utilize a laminin-511 scaffold, the interaction between laminin-511 and α6β1 integrin therefore partly compensates for Y-27632 (a ROCK inhibitor). A number of studies have indicated that α6β1-integrin is highly expressed and/or have described the role of this integrin in PSC adhesion [18–21] and self-renewal [22]. We examined the mRNA levels of integrin α6 and β1 expressed by hiPSCs when iMatrix-511 and collagen+MFGE8 were used as scaffolds.

**FIG. 3.**
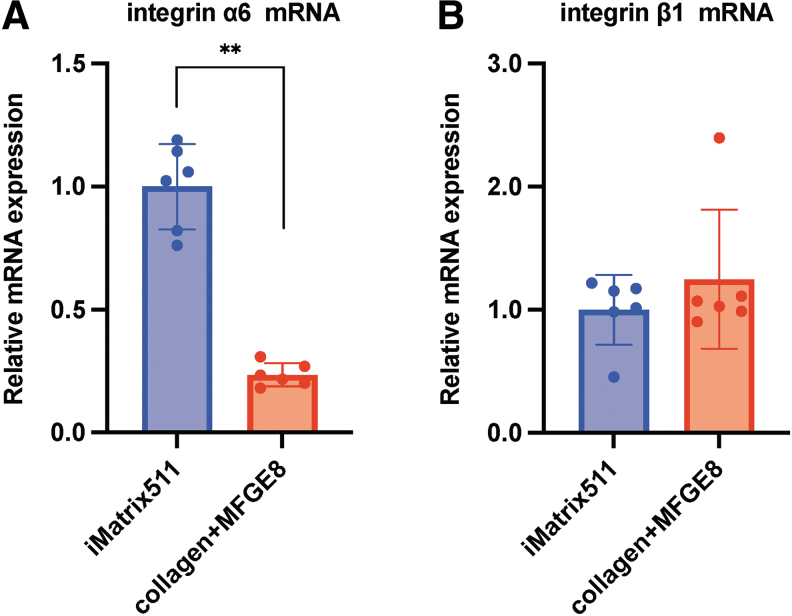
mRNA expression analysis. **(A)** The integrin α6 and **(B)** *integrin β1* mRNA expression from hiPSCs on iMatrix511 or collagen+MFGE8 was measured by real-time PCR. Values indicate the relative value obtained by converting the calculated value of iMatrix511 to 1 (*n* = 6). The data are presented as the mean ± SD: standard error. *P < 0.05, **P < 0.01. SD, standard deviation.

The results showed that the expression of integrin α6 was significantly decreased when collagen+MFGE8 was used as a scaffold material in comparison to iMatrix-511 (mean [standard deviation {SD}]: iMatrix-511 1.00 [0.42]; collagen+MFGE8, 0.23 [0.047]; **P < 0.01; *n* = 6) (Fig. 3A). On the other hand, the mRNA expression of integrin β1 was similar when iMatrix-511 and collagen+MFGE8 were used as scaffolds (mean [SD]: iMatrix-511, 1.00 [0.45]; collagen+MFGE8, 1.25 [0.57]; *n* = 6) (Fig. 3B). The α6β1 integrin on the cell membrane surface may decrease if trypsin is used to harvest cells.

### Fyn-p85-PI3K signaling

**FIG. 4.**
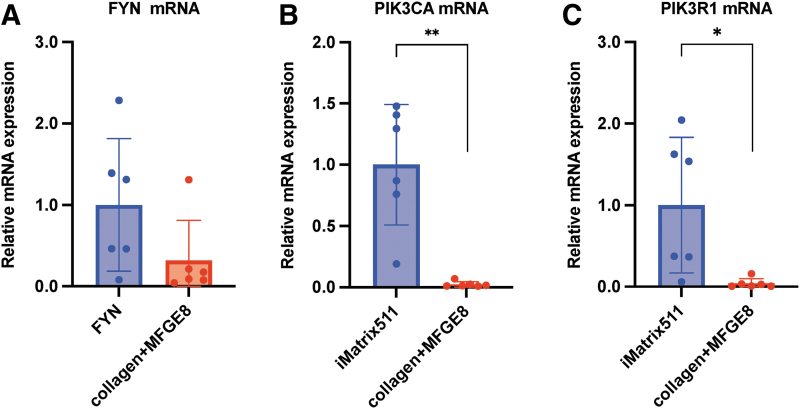
mRNA expression analysis. The **(A)** FYN, **(B)** PIK3CA, and **(C)** *PIK3R1* mRNA expression from hiPSCs on iMatrix511 or collagen+MFGE8 was measured by real-time PCR. Indicates the relative value obtained by converting the calculated value of iMatrix511 to 1 (*n* = 6). The data are presented as the mean ± SD: standard error. *P < 0.05, **P < 0.01.

Fyn, a proto-oncogene tyrosine-protein kinase encoded by the FYN gene [23], is associated with the p85 subunit of PI3K. There are few reports on the role of FYN in hiPSCs. We first examined whether or not hiPSCs express *FYN* mRNA and confirmed that hiPSC expresses *FYN* mRNA. The Fyn-RhoA-ROCK signaling cascade [24] has received attention as Fyn is directly associated with α6β1-integrin [25,26]. We examined the mRNA levels of FYN expressed by hiPSCs when iMatrix-511 and collagen+MFGE8 were used as scaffolds. The results showed that the expression of FYN was decreased when collagen+MFGE8 was used as a scaffold material in comparison to iMatrix-511 (mean [SD]: iMatrix-511, 1.00 [0.73]; collagen+MFGE8, 0.32 [0.50]; *n* = 6) (Fig. 4A).

Previous reports have shown that the PI3K/AKT signaling pathway has an essential role in the survival of iPSCs [27]. PI3K is a heterodimer consisting of catalytic subunits (p110α, p110β, and p110δ) and regulatory subunits (p85α, p55α, p50α, p85β, and p55γ). The catalytic subunit p110α is encoded by PIK3CA, and the regulatory subunit p85α is encoded by PIK3R1. This means that a decrease in the expression of PIK3CA or PIK3R1 leads to a weakening of the PI3K/AKT signaling pathway. Furthermore, we previously reported that the reduced expression of PIK3CA makes it extremely difficult for hiPSCs to survive [15]. The results showed that the expression of PIK3CA (Phosphatidylinositol-4,5-Bisphosphate 3-Kinase Catalytic Subunit Alpha; also called the p110α protein), which is a class of PI3K catalytic subunit [28,29], was significantly decreased when collagen+MFGE8 was used as a scaffold material in comparison to iMatrix-511 (mean [SD]: iMatrix-511, 1.00 [0.61]; collagen+MFGE8, 0.024 [0.02]; **P < 0.01; *n* = 6) (Fig. 4B).

The results showed that the expression of PIK3R1 (Phosphoinositide-3-Kinase Regulatory Subunit 1) was significantly decreased when collagen+MFGE8 was used as a scaffold material in comparison to iMatrix-511 (mean [SD]: iMatrix-511, 1.00 [0.79]; collagen+MFGE8, 0.04 [0.06]; *P < 0.05, *n* = 6) (Fig. 4C). These results showed that the scaffold material iMatrix-511 enhanced the PI3K/AKT pathway of hiPSCs.

### E-cadherin-catenin-Rac1-p120-P190 RhoGAP signaling

**FIG. 5.**
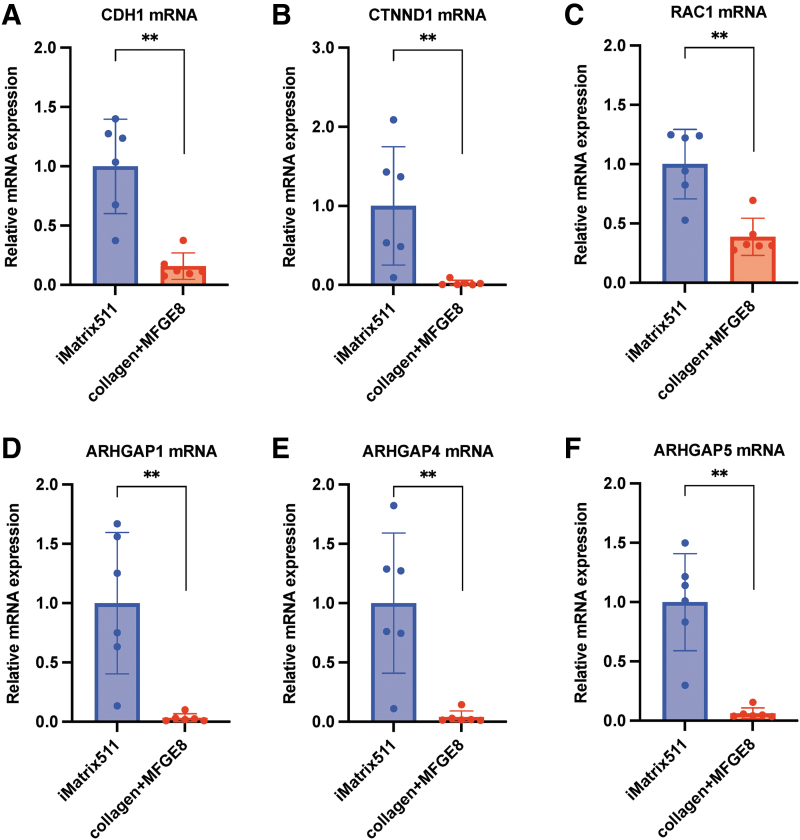
mRNA expression analysis. **(A)** CDH1, **(B)** CTNND1, **(C)** RAC1, **(D)** ARHGAP1, **(E)** ARHGAP4, and **(F)** *ARHGAP5* mRNA expression from hiPSC on iMatrix511 or collagen+MFGE8 was measured using real-time PCR. Values indicate the relative value obtained by converting the calculated value of iMatrix511 to 1 (*n* = 6). The data are presented as the mean ± SD: standard error. *P < 0.05, **P < 0.01.

Cadherin-1, which is also known as CAM 120/80 or epithelial cadherin (E-cadherin) or uvomorulin, is a protein that is encoded by the CDH1 gene in humans. E-cadherin, a Ca^2+^-dependent cell–cell adhesion molecule [30,31], is stabilized at the cell surface through its link—through β-catenin and α-catenin—to the cytoskeleton of actin. It is essential for the intercellular adhesion and colony formation of PSCs [32–35]. The loss of this E-cadherin-dependent intercellular adhesion can cause cell death [36]. The results showed that the expression of CDH1 was significantly decreased when collagen+MFGE8 was used as a scaffold material in comparison to iMatrix-511 (mean [SD]: iMatrix-511, 1.00 [0.38]; collagen+MFGE8, 0.16 [0.11]; **P < 0.01; *n* = 6) (Fig. 5A). The results showed that the scaffold material, iMatrix-511, significantly enhanced the E-cadherin expression of hiPSCs. Endocytosis and recycling regulate the level of E-cadherin at adherens junctions; activated Rac1 reduces E-cadherin endocytosis, thereby increasing the E-cadherin level on the cell surface and consequently promoting cell–cell adhesion.

The ectopic overexpression of E-cadherin also increases the survival of dissociated hPSCs [37]. However, dissociated hPSCs grown on E-cadherin-coated plates form membrane protrusions and show a lower survival rate. Taken together, E-cadherin interactions are not the sole factor affecting cell survival [36]. It appears that transcription factors for the expression of E-cadherin act downstream in various signaling pathways [eg, TGF-β, FGF2, nuclear factor κB (NFκB), and integrin cascades] [35].

The CTNND1 gene provides instructions for making a protein called p120-catenin, also known as delta 1 catenin. E-cadherin-mediated adhesion has been reported to stimulate PI3K/AKT signaling and to have an association with β-catenin signaling [38,39]. The promotion of cell survival by the PI3K/AKT pathway indicates that the Rho family GTPases have a wider role through p120-catenin [40,41]. p120-catenin may also influence cytoskeletal organization in the cytoplasm through the regulation of the opposing activities of Rho and Rac GTPase organization [42]. The results showed that the expression of CTNND1 was significantly decreased when collagen+MFGE8 was used as a scaffold material in comparison to iMatrix-511 (mean [SD]: iMatrix-511, 1.00 [0.71]; collagen+MFGE8, 0.025 [0.03]; **P < 0.01; *n* = 6) (Fig. 5B). Many of the activities of p120-catenin are associated with increased cellular proliferation and alteration of the cell cycle [43]. As a result, the use of iMatrix-511 as a scaffold material accelerates the increase in cell proliferation through the activation of p120-catenin.

Rho-GTPases, small G-proteins that mediate cellular proliferation and motility [44], are involved in regulating cell survival or death decisions. Functional interaction between Rac1, a small Rho family GTPase, and E-cadherin have been demonstrated to be responsible for the regulation of hPSC self-renewal [45,46]. The results showed that the expression of RAC1 was significantly decreased when collagen+MFGE8 was used as a scaffold material in comparison to iMatrix-511 (mean [SD]: iMatrix-511, 1.00 [0.28]; collagen+MFGE8, 0.39 [0.15]; **P < 0.01; *n* = 6) (Fig. 5C). According to previous reports, cadherin engagement can inhibit RhoA activity and activate Rac1 [47].

P190 RhoGAP (GTPase-activating protein) was demonstrated to be an important binding partner of p120 [48]. The RhoGAP domain is located at the C-terminal end of p190RhoGAP proteins. Both p190A and B mainly have catalytic activity toward RhoA [49,50]. The inactivation of RhoA by p190 RhoGAP regulates the spreading and migration of cells through the promotion of membrane polarity and membrane protrusion [51]. ARHGAP1, also known as RhoGAP, RhoGAP1, CDC42GAP, and p50rhoGAP, is officially named Ras homology (Rho) GTPase-activating protein 1, which is one of the key members of the RhoGAPs [52]. The results showed that the expression of ARHGAP1 was significantly decreased when collagen+MFGE8 was used as a scaffold material in comparison to iMatrix-511 (mean [SD]: iMatrix-511, 1.00 [0.57]; collagen+MFGE8, 0.03 [0.03]; **P < 0.01; *n* = 6) (Fig. 5D).

Rho GTPase-activating protein p115 or ARHGAP4 is encoded by the gene ARHGAP4 and is a member of the RHO GTPase-activating proteins (rhoGAP) family of proteins. It has been reported that ARHGAP4 regulates the β-catenin pathway. The results showed that the expression of ARHGAP4 was significantly decreased when collagen+MFGE8 was used as a scaffold material in comparison to iMatrix-511 (mean [SD]: iMatrix-511, 1.00 [0.56]; collagen+MFGE8, 0.039 [0.049]; **P < 0.01; *n* = 6) (Fig. 5E).

The ARHGAP5 gene provides instructions for making a protein called Rho GTPase-activating protein 5 (Rho-type GTPase-activating protein 5) (p190-B). The results showed that the expression of ARHGAP5 was significantly decreased when collagen+MFGE8 was used as a scaffold material in comparison to iMatrix-511 (mean [SD]: iMatrix-511, 1.00 [0.39]; collagen+MFGE8, 0.06 [0.04]; **P < 0.01; *n* = 6) (Fig. 5F). These results showed that the expression of p190 RhoGAP, which deactivates RhoA, was significantly higher in hiPSCs cultured with iMatrix-511 as scaffold material.

### RhoA-ROCK signaling

**FIG. 6.**
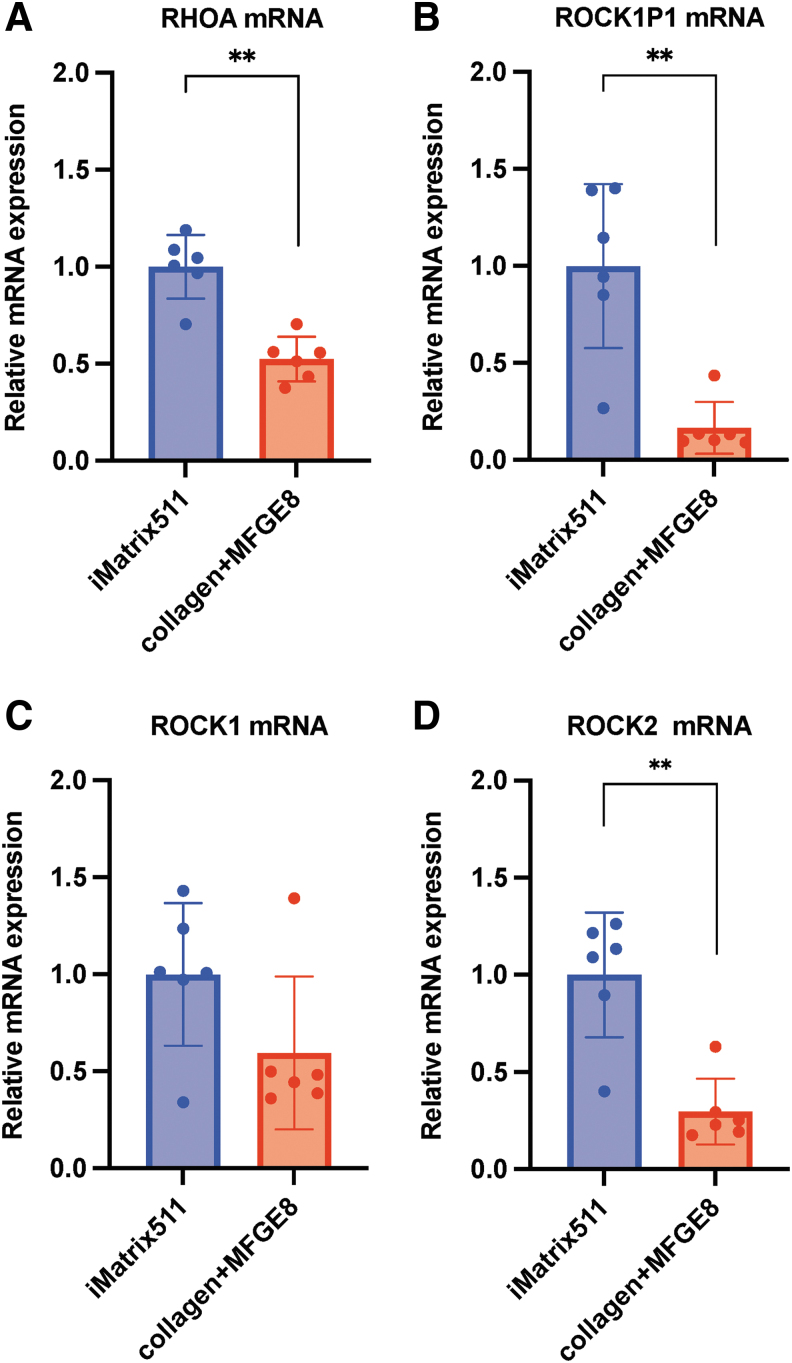
mRNA expression analysis. The **(A)** RHOA, **(B)** ROCK1P1, **(C)** ROCK1, and **(D)** *ROCK2* mRNA expression from hiPSCs on iMatrix511 or collagen+MFGE8 was measured by real-time PCR. Values indicate the relative value obtained by converting the calculated value of iMatrix511 to 1 (*n* = 6). The data are presented as the mean ± SD: standard error. *P < 0.05, **P < 0.01.

RhoA is a 22-kDa small GTPase, a member of the Ras superfamily and Rho subfamily of GTPases. The results showed that the expression of RhoA was significantly decreased when collagen+MFGE8 was used as a scaffold material in comparison to iMatrix-511 (mean [SD]: iMatrix-511, 1.00 [0.16]; collagen+MFGE8, 0.52 [0.11]; **P < 0.01; *n* = 6) (Fig. 6A).

The effect of ROCK1P1 (Rho-associated coiled-coil containing protein kinase 1 pseudogene 1) on hiPSCs is unknown, but its mRNA expression was observed. The results showed that the expression of ROCK1P1 was significantly decreased when collagen+MFGE8 was used as a scaffold material in comparison to iMatrix-511 (mean [SD]: iMatrix-511, 1.00 [0.40]; collagen+MFGE8, 0.17 [0.13]; **P < 0.01; *n* = 6) (Fig. 6B).

The small molecule Rho-associated kinase (ROCK) proteins consist of two subunits, ROCK1 and ROCK2. Rho kinases (ROCK1 and ROCK2) function downstream of the small GTPase RhoA to drive actomyosin cytoskeletal remodeling [53]. Cell adhesion, cell morphology, and cytoskeletal tension regulate the activation of ROCK by RhoA [54]. The ROCK inhibitor Y-27632, or a combination of ROCK inhibitors, has been shown to enhance hPSC survival after passage as single cells [55,56]. The cell–cell interaction mediated by ROCK inhibitor seems to be associated with the stabilization of the cell surface by E-cadherin. The results showed that the expression of ROCK1 was decreased when collagen+MFGE8 was used as a scaffold material in comparison to iMatrix-511 (mean [SD]: iMatrix-511, 1.00 [0.35]; collagen+MFGE8, 0.59 [0.38]; *n* = 6) (Fig. 6C). The results showed that the *ROCK1* mRNA expression of ROCK1 was similar when iMatrix-511 and collagen+MFGE8 were used as scaffolds.

In a prior experiment with *n* = 3 specimens, hiPSCs cultured on the scaffold material collagen+MFGE8 showed significantly higher *ROCK1* mRNA expression than hiPSCs cultured on iMatrix-511 (data not shown). In this experiment conducted with *n* = 6 specimens, hiPSCs cultured on iMatrix-511 had their samples collected on day 5, whereas hiPSCs cultured on collagen+MFGE8 had their samples collected on day 8 due to slow cell growth. The expression of *ROCK1* mRNA seems to be affected by the timing of sample collection after cell seeding.

The results showed that the expression of ROCK2 was significantly decreased when collagen+MFGE8 was used as a scaffold material in comparison to iMatrix-511 (mean [SD]: iMatrix-511, 1.00 [0.31]; collagen+MFGE8, 0.30 [0.16]; **P < 0.01; *n* = 6) (Fig. 6D). Since the ROCK inhibitor Y-27632 inhibits targets of both ROCK1 and ROCK2, it was assumed that the effect of iMatrix-511 would reduce the levels of both ROCK1 and ROCK2, but in fact, when the expression of *ROCK2* mRNA was checked, iMatrix-511 was found to promote the expression of *ROCK2* mRNA. This result suggests that only ROCK1—and not ROCK2—affects the cell death of hiPSCs.

### Western blotting

Western blotting was performed for various factors involved in the Fyn-RhoA-ROCK signaling pathway. Unfortunately, no experimental results are available for integrin α6, ROCK-1, ROCK-2, PI3-kinase (p85α), P190-A RhoGAP, and P190-B RhoGAP because nonspecific bands were identified.

**FIG. 7.**
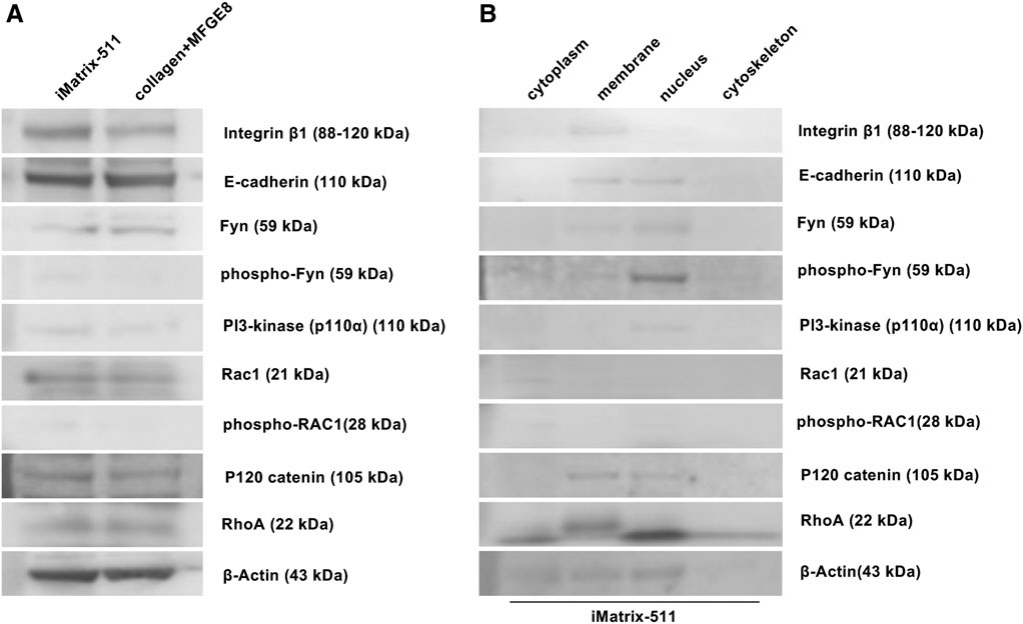
Western blotting. **(A)** Western blot assay image of E-Cadherin, integrin α6, integrin β1, Fyn, phospho-Fyn, Rac1, phospho-Rac1, p120 catenin, PI3-kinase (p85α), PI3-kinase (p110α), p190-A RhoGAP, p190-B RhoGAP, RhoA, ROCK-1, ROCK-2, and β-actin bands photosensitively detected on the membrane. **(B)** Proteins of iPSCs cultured on iMatrix-511 were fractionated into four groups (cytoplasm, plasma membrane, nucleus, and cytoskeleton). Western blot assay image of E-Cadherin, integrin α6, integrin β1, Fyn, phospho-Fyn, Rac1, phospho-Rac1, p120 catenin, PI3-kinase (p85α), PI3-kinase (p110α), p190-A RhoGAP, p190-B RhoGAP, RhoA, ROCK-1, ROCK-2, and β-actin bands photo-sensitively detected on the membrane of each lanes (fractionated into cytoplasm, plasma membrane, nucleus, and cytoskeleton). The samples flowed into each lane (10 μl) of the membrane.

First, we investigated the mechanism by which iMatrix-511 activates PI3K through integrin α6β1 and Fyn for the Fyn-RhoA-ROCK signaling pathway. The integrin β1 band was detected in whole cell proteins of iPSCs cultured on iMatrix-511 and collagen+MFGE8 (Fig. 7A). When the proteins of iPSCs cultured on iMatrix-511 were fractionated into cytoplasm, plasma membrane, nucleus, and cytoskeleton, the integrin β1 band was detected in the plasma membrane. A narrow band of phosphorylated Fyn was detected in iPSCs cultured on iMatrix-511 in whole cells. In contrast, no band of phosphorylated Fyn was detected in iPSCs cultured on collagen+MFGE8 in whole cells (Fig. 7A). When proteins were fractionated into cytoplasm, plasma membrane, nucleus, and cytoskeleton, iPSCs cultured on iMatrix-511 showed thin bands of Fyn in the plasma membrane and nucleus, whereas thin bands of phosphorylated Fyn were detected in the plasma membrane, and thick bands were detected in the nucleus (Fig. 7B).

The band of PI3-kinase (p110α) was detected as a thin band in both iPSCs cultured on iMatrix-511 and all protein samples obtained from iPSCs cultured on collagen+MFGE8 (Fig. 7A). The band of PI3-kinase (p110α) from iPSCs cultured on iMatrix-511 in whole cells showed a narrow band in the nucleus (Fig. 7B). In conclusion, iMatrix-511 phosphorylates Fyn on the cell surface through integrin α6β1. Phosphorylated Fyn is translocated to the nucleus, and nuclear translocation of PI3K seems to occur together with the phosphorylation of Fyn.

Both E-cadherin and P120 catenin bands were detected in total proteins of iPSCs cultured on iMatrix-511 and collagen+MFGE8 (Fig. 7A). When the proteins of iPSCs cultured on iMatrix-511 were fractionated into four groups (cytoplasm, plasma membrane, nucleus, and cytoskeleton), E-cadherin and P120 catenin were both detected in the plasma membrane and nucleus (Fig. 7B).

Rac1 bands were detected in total proteins of iPSCs cultured on iMatrix-511 and collagen+MFGE8 (Fig. 7A). No bands of phospho-RAC1 were detected in total protein of iPSCs cultured in collagen+MFGE8, and only in total protein of iPSCs cultured with iMatrix-511 was a thin band of phospho-RAC1 detected (Fig. 7A). When the proteins of iPSCs cultured on iMatrix-511 were fractionated into four groups (cytoplasm, plasma membrane, nucleus, and cytoskeleton), Rac1 and phospho-RAC1 were both detected in the cytoplasm. Given the above, it appears that iMatrix-511 promotes phosphorylation of RAC1 in the cytoplasm of iPSCs. In addition, E-cadherin and P120 catenin on the plasma membrane can be activated by phospho-RAC1.

RhoA bands were detected in total proteins of iPSCs cultured on iMatrix-511 and collagen+MFGE8 (Fig. 7A). When the proteins of iPSCs cultured on iMatrix-511 were fractionated into four groups (cytoplasm, plasma membrane, nucleus, and cytoskeleton), RhoA was detected in the membrane (Fig. 7B).

### Proteome analyses

**FIG. 8.**
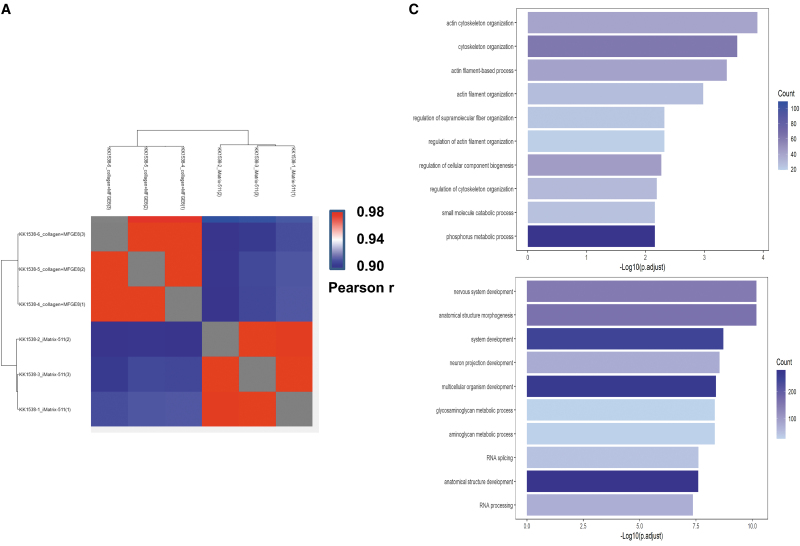

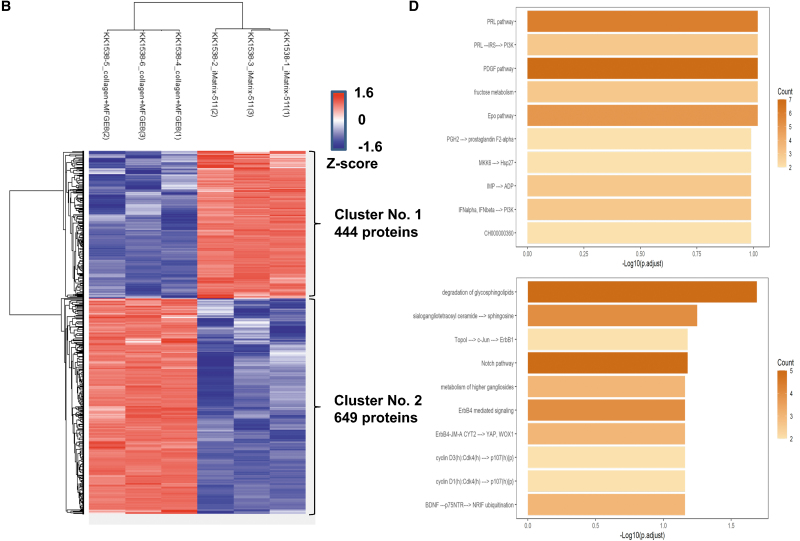
Standard DIA proteome analysis findings. **(A)** The correlation coefficient (Pearson *r*) between samples is calculated from the overall quantitative values, and a heatmap is created to cluster and present the differences between samples. **(B)** Proteins with *P* < 0.05 (two groups: *T*-test) were selected if the mean value of each group varied more than twofold, and the difference between groups were *P* < 0.05 (two groups: *T*-test). Each quantitative value was converted to a *Z*-score, and a heatmap was created. **(C)** The results of the GO analysis of increased protein groups in cluster 1 (*upper panel*) or increased protein groups in cluster 2 (*lower panel*), showing the bar graph of the top 10 in the result of Biological Process is shown. **(D)** The results of the Pathway analysis in the increased protein groups in cluster 1 (*upper panel*) or increased protein groups in cluster 2 (*lower panel*), showing the bar graph of the top 10 in the result of Pathway analysis is shown. DIA, data-independent acquisition.

Proteins were extracted from samples of hiPSCs cultured on iMatrix-511 and collagen+MFGE8 scaffolds, fragmented into peptide fragments by digestive enzymes, and subjected to a DIA analysis by LC-MS. The obtained data were analyzed using the DIA proteome analysis software program (Scaffold DIA) to identify proteins and peptides with both Peptide false discovery rate (FDR) and Protein FDR <1% and to calculate quantitative values. The identification and quantification results from the analysis software program (Scaffold DIA) are shown. Protein quantification values were calculated from the DIA analysis data, with “0” meaning that the protein could not be detected (missing value). Normalization was performed based on the median of the quantitative values (Supplementary Table S1). The quantitative values of this DIA analysis datum were Log2 transformed, and missing values (quantitative values of 0) were randomly assigned at low values that were below the detection limit. A heatmap was created by determining the correlation coefficient (Pearson *r*) between samples from the overall quantitative values, and differences between samples were clustered (Fig. 8A). Next, proteins were selected that satisfied the following conditions: (1) the mean value of each group varied by a factor of ≥2, and (2) the difference between groups was *P* < 0.05 (two groups: *T*-test, ≥3 groups: analysis of variance).

Each quantitative value was converted to a *Z*-score, and a heatmap was created (Fig. 8B). The 444 proteins in cluster 1 comprised a group in which the protein expression by iPSCs cultured on iMatrix-511 was higher than that by iPSCs cultured on collagen+MFGE8. A list of the 444 proteins in cluster 1 is shown in Supplementary Table S2. The 649 proteins in cluster 2 comprised group in which the protein expression by iPSCs cultured on collagen+MFGE8 was higher than that by iPSCs cultured on iMatrix-511. A list of the 649 proteins in cluster 2 is shown in Supplementary Table S3.

The protein groups with increased protein quantification in cluster 1 (average protein quantification in cluster 1) increased more than twofold compared with cluster 2, and the difference between the groups was *P* < 0.05 (two groups: *T*-test). Analyses by GO, Pathway, and an Upstream analysis of GeneXplain were performed. iPSCs cultured on iMatrix-511 showed a greater expression of a group of proteins related to the regulation of the cytoskeleton, mainly actin, than iPSCs cultured on collagen+MFGE8 (Fig. 8C, upper panel) (Supplementary Table S4). iPSCs cultured on iMatrix-511 showed a greater expression of a group of proteins related to the regulation of the PI3K signaling than iPSCs cultured on collagen+MFGE8 (Fig. 8D, upper panel) (Supplementary Table S6). These results indicate that iMatrix-511 activates PI3K signaling, which is reported to be essential for the survival [15] of iPSCs. In addition, the mechanism of actin, a cytoskeletal protein, was shown to be active.

The protein groups with increased protein quantification in cluster 2 (the average protein quantification in cluster 2) increased more than twofold compared with cluster 1 and the difference between the two groups was *P* < 0.05 (two groups: *T*-test), analyzed by GO and Pathway, and Upstream analysis of GeneXplain were performed. The iPSCs cultured in collagen+MFGE8 showed higher expression of a group of proteins related to neural differentiation, glycosaminoglycan metabolism, aminoglycan metabolism, RNA splicing, and RNA processing compared with iPSCs cultured in iMatrix-511 (Fig. 8C, lower panel) (Supplementary Table S5). The iPSCs cultured in collagen+MFGE8 showed a higher expression of a group of proteins related to degradation of glycosphingolipids, sialogangliotetraosyl ceramide, the Notch pathway, YAP, cyclin D3, and cyclin D1 than iPSCs cultured in iMatrix-511 (Fig. 8D, lower panel) (Supplementary Table S7).

These results indicate that Notch signaling of iPSCs and YAP are activated in the absence of iMatrix-511. They also indicate that neural developmental mechanisms are active. The iMatrix-511 has been previously reported to inhibit YAP [57]. Notch signaling is known to be involved in many differentiation processes, including neural, hematopoietic, vascular, and somatic differentiation.

## Discussion

While xeno-free media, which are alternatives to animal serum, contain no animal-derived components, they may contain human-derived components. A combination of collagen IV, fibronectin, laminin, and vitronectin can be used instead of Matrigel to derive and expand hPSCs under specific culture conditions [58]. However, for culture materials that can be used to produce clinical iPSCs, to give top priority to safety, it is necessary to select materials that conform to the Japanese Standards for Biological Ingredients (JSBI). The JSBI stipulate measures to be taken to ensure the quality, efficacy, and safety of pharmaceuticals, quasi-drugs, cosmetics, medical devices, and regenerative medicine when raw materials used in these products are derived from other living organisms other than humans and plants (https://www.pmda.go.jp/files/000204341.pdf).

In 2008, it was reported that the self-renewal ability of mouse ES cells was enabled by the use of laminin-511, but not laminin-332, -111, or -411, as a scaffold material [16]. In 2010, it was reported that long-term self-renewal of human pluripotent cells was enabled by using recombinant protein laminin-511 as a scaffold material [17].

Laminin 511, a recombinant protein from CHO cells, was developed in 2014 as a product (iMatrix-511: Nippi, Tokyo, Japan) that meets the safety standards for culture materials used for clinical cell production under Good Manufacturing Practice manufacturing control and the JSBI by incorporating sterility and virus negation tests into the manufacturing process. The product was developed in 2014. The iMatrix-511 is used as a scaffold material to produce master cell banks, which are the most fundamental cell material for the current production of iPSCs for clinical use [1]. The laminin-511 scaffold can bind the α3β1, α6β1, and α6β4 integrins. In a feeder cell-free culture system, it can be applied to maintain hPSCs in an undifferentiated state under serum and xeno-free conditions [2–6].

Laminin isoforms, laminin-511 and -521, are expressed by human embryonic stem cells (hESCs) [59]. Therefore, before the development of laminin-511 as a scaffold material, ES cell and iPSC researchers tried to prevent cell death by passaging hiPSCs in clumps by physically dissociating the colonies or weakly activating trypsin. By passaging hiPSCs in clumps, ES cell and iPSC researchers were able to manipulate hiPSCs without cleaving α6β1 integrin-Fyn-RhoA-ROCK signaling.

In addition, it was possible to passage hiPSCs without using ROCK inhibitors when hiPSCs colonies became dense. However, to systematically cultivate high-quality hiPSCs using a culture method that relies on the state of viable cells, ES cell and iPSC researchers needed to be skilled in hiPSC colony mass size and colony density. However, with the development of laminin-511, the difficulty of culturing hiPSCs has been greatly simplified, the stable supply of high-quality hiPSC has become possible, and the hiPSC manufacturing process has reached a technological level that is suitable for industrialization.

## Conclusion

Binding of α6β1 with laminin-511 and interaction with E-cadherin stimulates Fyn-Rhoa-ROCK signaling to deactivate ROCK signaling, which indicates that the signaling pathways induced by laminin-511 scaffolds are dependent on PI3K signaling through E-cadherin-mediated cell–cell adhesion, and further, demonstrate an association between the Fyn-RhoA-ROCK signaling cascade and α6β1 integrin-laminin-511 adhesion. Therefore, the ROCK inhibitor Y-27632 is unnecessary when the expression of ROCK1 is suppressed by the effect of the scaffold material iMatrix511. However, if the Fyn-Rhoa-ROCK signaling in the cells is broken down and ROCK1 is activated by exposure to trypsin, Y-27632 must be added to the culture medium during the hiPSC culture period, until Fyn-Rhoa-ROCK signaling is restored.

## Supplementary Material

Supplemental data

Supplemental data

Supplemental data

Supplemental data

Supplemental data

Supplemental data

Supplemental data
